# Heterogeneity of stimulus-specific response modification—an fMRI study on neuroplasticity

**DOI:** 10.3389/fnhum.2014.00695

**Published:** 2014-09-08

**Authors:** Jacob Lahr, Jessica Peter, Michael Bach, Irina Mader, Christoph Nissen, Claus Normann, Christoph P. Kaller, Stefan Klöppel

**Affiliations:** ^1^Freiburg Brain Imaging, University Medical Center FreiburgFreiburg, Germany; ^2^Department of Psychiatry and Psychotherapy, University Medical Center FreiburgFreiburg, Germany; ^3^Department of Neurology, University Medical Center FreiburgFreiburg, Germany; ^4^Laboratory for Biological and Personality Psychology, Department of Psychology, University of FreiburgFreiburg, Germany; ^5^Eye Center, University Medical Center FreiburgFreiburg, Germany; ^6^Department of Neuroradiology, University Medical Center FreiburgFreiburg, Germany

**Keywords:** VEP, LTD (long term depression), LTP (long term potentiation), neuronal plasticity, fMRI (functional magnet resonance imaging), habituation

## Abstract

Long-term potentiation (LTP) is a key element of synaptic plasticity. At the macroscopic level, similar effects can be induced in the human brain using repetitive stimulation with identical stimuli. High-frequency stimulation (HFS) can increase neuronal responses whereas low-frequency stimulation may produce the opposite effect. Optimal stimulation frequencies and characteristics for inducing stimulus-specific response modification (SRM) differ substantially from those applied to brain tissue slices but have been explored in recent studies. In contrast, the individual manifestation of this effect in terms of its spatial location and extent are unclear. Using functional magnetic resonance imaging (fMRI) in 18 subjects (mean age 25.3 years), we attempted to induce LTP-like effects by HFS with checkerboard flashes at 9 Hz for 120 s. As expected, flashes induced strong activation in primary and secondary visual cortices. Contrary to our expectations, we found clusters of decreased activations induced by pattern flashes after HFS in the primary and secondary visual cortices. On the level of the individual subject, some showed significantly increased activations in the post-HFS session while the majority showed significant decreases. The locations of areas showing altered activations before and after HFS were only partly overlapping. No association between location, extent and direction of the HFS-effect was observed. The findings are unexpected in the light of existing HFS-studies, but mirror the high inter-subject variability, concerning even the directionality of the induced effects shown for other indices of LTP-like plasticity in the human brain. As this variability is not observed in LTP at the cellular level, a better understanding of LTP-like mechanisms on the macroscopic level is essential for establishing tools to quantify individual synaptic plasticity *in-vivo*.

## Introduction

The brain's ability to adapt is referred to as neuronal plasticity. Neuronal plasticity is important in the context of learning and reorganization of the brain when facing acute (e.g., stroke) or continuous damage (e.g., neurodegeneration, aging).

Currently, synaptic long-term potentiation (LTP) is among the best understood molecular mechanisms underlying neuronal plasticity. It is characterized by a long-term increase of synaptic transmission following repetitive stimulation. LTP can be induced by tetanic stimulation at high frequencies, or by associative pre- and postsynaptic stimulation (Cooke and Bliss, 2006). LTP has been studied extensively on a cellular and molecular level in animals, mainly in the hippocampus (Bliss and Lømo, 1973) but also in the visual (Komatsu et al., 1981) and somatosensory cortex (Fox, 2002) as well as the spinal cord (Ji et al., 2003). In human slice preparations obtained from brain surgery, LTP activity has been demonstrated in hippocampal (Beck et al., 2000) and temporal lobe (Chen et al., 1996) specimens. LTP is complemented by a mechanism called long-term depression (LTD) which reduces synaptic efficacy. *In vitro*, tetanic stimulation at relatively high frequencies (>30 Hz) induces LTP, whereas trains of stimulation at low frequencies (<10 Hz) induce LTD. For associative stimulation, the resulting effect depends on the temporal order of pre- and post-synaptic action potentials (Markram et al., 1997; Bi and Poo, 1998).

Several approaches to measure LTP-like effects non-invasively in intact human brains have been presented. As the exact nature of the measured effects in the following paradigms is unknown, these are often referred to as “LTP-like.” Aiming for LTP-like effects, some studies induced stimulus-specific response potentiation (SRP) by repeatedly presenting the identical visual or auditory stimulus and compared the brain's response before and after stimulation using electroencephalography (EEG) (Clapp et al., 2005a; Teyler et al., 2005; Normann et al., 2007; Beste et al., 2012), or functional magnetic resonance imaging (fMRI) (Clapp et al., 2005b; Zaehle et al., 2007). As its name indicates, SRP essentially refers to response potentiation using high-frequency stimulation (HFS), however, some studies have demonstrated response depression using the same stimuli at lower stimulation frequencies (Teyler et al., 2005; Beste et al., 2011)—thus stimulus-specific response modification (SRM) may be an appropriate broader term. Previous studies focused on similarities between SRM and LTP: The administration of an NMDAR-antagonist blocks visual SRP in rodents (Heynen and Bear, 2001; Clapp et al., 2006). In addition, SRP fulfills characteristics of LTP such as specificity (Normann et al., 2007; Ross et al., 2008) and stability for more than 1 h (Beste et al., 2011). LTP-like effects were also demonstrated for auditory stimulation in an fMRI (Zaehle et al., 2007), and in an EEG study (Clapp et al., 2005a), which suggests that LTP-like mechanisms exist globally in the brain.

The only existing SRM study in the visual system using fMRI (Clapp et al., 2005b) in 10 subjects reported effects only at the group level and after integrating across both hemispheres. Using fMRI, we aimed to analyze the individual manifestation of checkerboard-flashes induced SRM in terms of strength, spatial location, and extent of areas involved. Identifying associations between these three factors may help to improve our understanding of the underlying mechanisms and help to establish SRM as a tool to characterize individual plasticity. We focused on the visual system because it is a well accessible system where SRM at the group level has been studied most extensively using EEG in humans (e.g., Teyler et al., 2005; Normann et al., 2007; Beste et al., 2012). Signal changes were located using fMRI as its spatial resolution is superior compared to EEG.

Taking stimulation parameters (checkerboard stimulus, stimulation frequency of 9 Hz, duration of 2 min) from previous studies on response potentiation in the visual cortex as a starting point (Clapp et al., 2005b; Teyler et al., 2005), we aimed to analyze the inter-individual variability of LTP-like effects, as shown for other LTP-like effects (e.g., using transcranial magnetic stimulation; Müller-Dahlhaus et al., 2008). Notably the stimulation frequency of 9 Hz was chosen because it showed a substantial LTP-like effect in previous studies (Clapp et al., 2005b; Teyler et al., 2005), which was explained by potential increasing frequencies during neuronal processing. In line with previous work, we primarily focused on primary (V1) and secondary (V2) visual cortices.

## Methods

Eighteen healthy volunteers aged 18–38 years (mean: 25.3 ± 3.00 years, 6 female, 15 right-handed) participated in this study. Subjects had no history of psychiatric or neurological diseases and were not under medication at the time of the experiment. To avoid daytime specific effects, all experiments were performed in the afternoon. Written informed consent was obtained from all subjects prior to the experiments. The study protocol was approved by the ethics commission of the University Medical Center Freiburg (approval #227/12), in agreement with the Declaration of Helsinki.

### SRM paradigm

**Figure 1:**
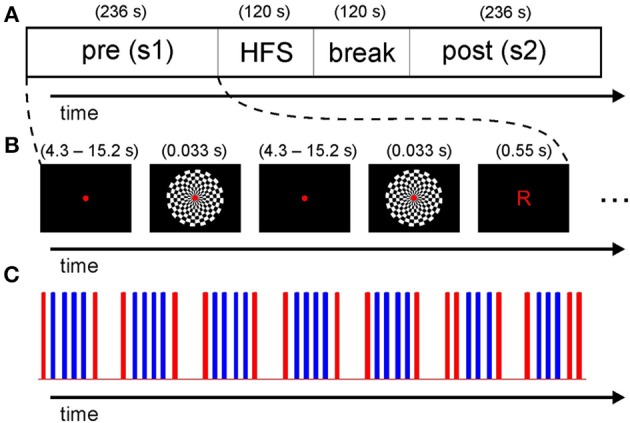
**Timing of the overall experiment**. **(A)** Subjects were presented with a pre-HFS session of checkerboard flashes and catch trials, followed by a high frequency stimulation (HFS) block and a 2 min break before the post-HFS session. **(B)** Timing of the individual stimuli. The subjects were required to fixate a red dot. Checkerboards and letters were presented at varying rates for optimal design efficiency. **(C)** The timing of all stimuli in one session is represented as a stick function. The checkerboard flashes are displayed in blue and the letters in red (see text for details on timing).

As mentioned in the introduction, we adapted existing paradigms. In contrast to Clapp et al. (2005b), checkerboard stimuli were shown to both hemispheres simultaneously as we were not interested in hemisphere specific effects. In addition, letters were presented in pseudorandomized intervals as catch trials to ensure constant focus throughout the experiment. Further, the duration was shortened and the timing of the probe stimulus presentation in the identical pre- and post-HFS sessions was optimized in terms of fMRI design efficiency using a genetic algorithm approach (Wager and Nichols, 2003). Most importantly, by avoiding long intervals between blocks of the same type, we sought to minimize signal loss due to subsequent high-pass filtering (see below). The overall time-course of the experiment is depicted in Figure 1A. Stimuli were presented with Presentation software (Version 16.1, Neurobehavioral Systems) using a video projector (frame rate: 60 Hz) and a screen in the bore of the scanner, viewed through a mirror mounted onto the head coil. The subjects received task instructions outside the scanner.

### Pre- and post-HFS sessions

For the probe sessions, the design resulting from optimizing efficiency nearly matched a typical block design (see Figures 1B,C for an illustration). The interval between two flashes in the short blocks of three or four flashes varied slightly between 4.0 and 4.9 s. Per session, 26 presentations of the checkerboard (duration: 33 ms), subtending a visual angle of 10.3° (0.77° for each field of the board) were shown. In an additional attention condition, 16 presentations of letters (“R” and “L,” duration: 550 ms, visual angle: 0.65°) were shown as catch trials (mean 15.2 s, median 12.9 s; range 4.3–21.5 s; see Figure 1B) similar to a related EEG study (Normann et al., 2007). The subjects were required to respond by pressing buttons with the index (“l”) or middle (“r”) finger of the right hand. We specifically did not instruct subjects to press buttons as fast as possible to avoid performance effects across the task. We displayed a small but clearly visible red dot in the middle of the screen for the whole duration of the task, except when letters were presented.

### HFS period

The HFS period consisted of a 2 min presentation of checkerboard flashes at a rate of 9 Hz (duration per checkerboard: 33 ms as above). Additionally, eight letters were presented as catch trials during the HFS period. Before the post-session, a pause of 2 min without visual stimulation was included to account for potential visual aftereffects (see Supplementary Figure S1 for the exact time course during the HFS period).

### Data acquisition

MRI was performed on a 3T whole-body scanner (Siemens TIM Trio) equipped with a 12-channel head coil. A T1-weighted whole-brain image was acquired as high-resolution structural reference for normalization and to exclude abnormalities (*TE*: 2.15 ms, *TR*: 2200 ms, flip angle: 12°, matrix size: 256 × 256 × 176, voxel-size: 1 × 1 × 1 mm). For fMRI, T2^*^-weighted gradient-echo echo-planar images (EPI) covering 192 × 192 × 81 mm were collected with the following parameters: *TE*: 32 ms, *TR*: 1920 ms, flip angle: 75°, matrix size: 80 × 80 × 28, voxel-size: 2.4 × 2.4 × 2.88 mm, gap: 20%, descending sequence, individually tilted to comprise the occipital cortex and the thalamus. Images were acquired continuously through probe and HFS periods. During reconstruction, scans were corrected for motion and distortion artifacts, based on a reference measurement (Zaitsev et al., 2006). Additionally, an EPI with identical parameters, but full brain coverage was acquired to aid coregistration with the individual T1-weighted image.

### Image processing

Two times 123 volumes, corresponding to the pre- and post-HFS probe sessions, were extracted from the continuously acquired stack and analyzed as two separate sessions. Images acquired during the HFS period were analyzed separately. Data analysis was performed using standard procedures from SPM8 (http://www.fil.ion.ucl.ac.uk). The volumes were spatially realigned and normalized to the Montreal Neurological Institute (MNI) reference brain, using the normalization parameters estimated during segmentation and normalization of the coregistered T1 anatomical scan (Ashburner and Friston, 2005). Eight-millimeter Gaussian smoothing was applied to reduce noise, and to reduce inter-subject differences.

### Statistical analysis

At the single subject level, the conditions “checkerboard flashes” and “letters” were modeled as regressors in each session. Additionally, the movement parameters obtained at the realignment step were included as regressors. The onsets and durations of the stimuli were convolved with the canonical hemodynamic response function. Multiple regression coefficients were calculated at each voxel. Of primary interest was the comparison of responses to checkerboard flashes before and after HFS, assessed via *t*-tests.

A parametric modulator was added to test for linear changes of the response to the flashes during the pre and post-HFS sessions (e.g., de- or increases of the fMRI signal with repetitive low frequency stimulation), this removes a linear component from the variance explained by the main regressor (i.e., visual stimulation). We focused on decreases during the pre- and post-HFS sessions, as this was reported in a previous EEG study (Teyler et al., 2005), increases are reported for completeness. To test if the HFS period for flashes had any effect on fMRI signal related to letters, we also compared those before and after the HFS period. As a study in mice found LTP-effects capable of inducing global changes in brain activity (Canals et al., 2009), we report between-session analyses across the whole field of view. Some analyses were restricted to the visual cortex (V1/BA17 and V2/BA18) using a mask based on cortical probability maps (Amunts et al., 2000).

At the multi-subject level, the respective individual parameter maps (i.e., difference between the parameter estimates of the general linear model for the pre-HFS and post-HFS session in the single subject analyses) were entered into one-sample *t*-tests. If not stated otherwise, statistical estimations were reported after correction for multiple comparisons at the cluster level as done by Clapp et al. (2005b). To this end, we applied a significance level of 0.01 uncorrected for multiple testing at the voxel level and *p* < 0.05 at the cluster level using the family-wise error correction (FWE; correction method not specified by Clapp and colleagues). Significant clusters were characterized by their peak-coordinate in MNI space and their cluster extent. To illustrate spatial extent and heterogeneity of individual effects of potentiation, we combined all binarized clusters within the mask of the visual cortex, significant at the individual subject level in one figure.

To illustrate signal changes during the HFS period and the probe sessions, we plotted the signal change in primary visual cortex, defined by a sphere (radius: 8 mm) around the peak voxel obtained from the main effect of flashes across both sessions in all subjects (*x, y, z*: [12, −94, 2], MNI-coordinates).

## Results

The behavioral data analyses revealed that the subjects correctly responded to 98.2% (range: 90–100%) of the catch trials (average response time: 659 ms range: 566–902 ms, standard deviation: 101 ms). There was no difference between the response time of the pre- and the post-HFS session [mean response time pre-HFS: 651 ms (*SD*: ±94 ms) post-HFS: 655 ms (*SD*: ±119 ms)].

**Figure 2:**
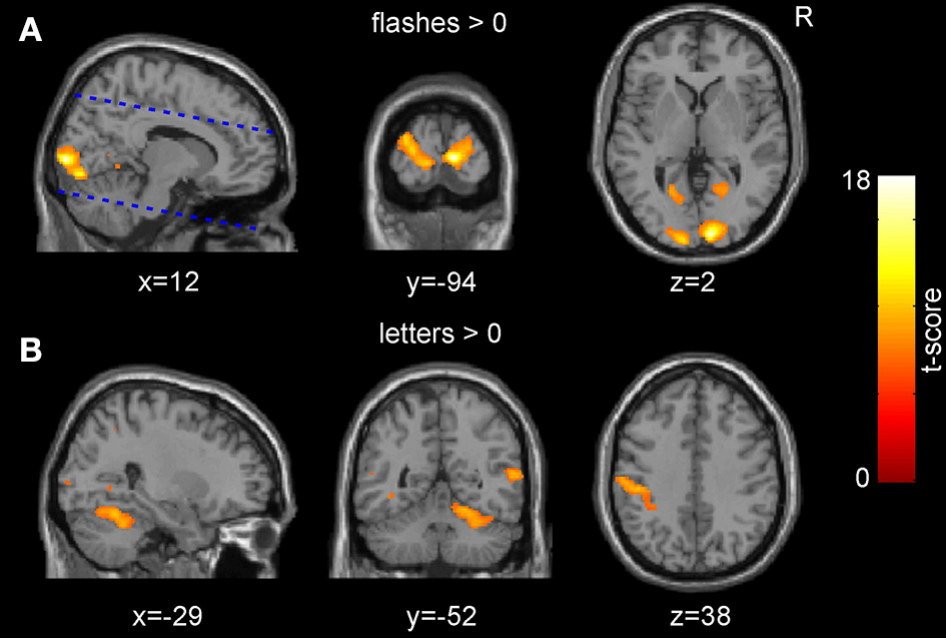
**Group-level main effects across both sessions**. **(A)** The presentation of the pattern flashes resulted in a significant activation in the occipital cortices. The dashed blue lines indicate the borders of the acquired field of view. **(B)** The presentation of the letters and subsequent responses resulted in an activation of several clusters including the somatosensory cortices (*p* < 0.05, FWE corrected for multiple comparisons at the voxel level).

The presentation of the checkerboard flashes induced a large significant occipital activation predominantly covering the visual cortices (peak voxel at [12, −94, 2], V1: 90%, V2: 10% according to previously published probabilistic atlas (Amunts et al., 2000; Figure 2A). For the presentation of letters and subsequent button presses, several clusters of activation were identified including those parts of the somatosensory system that were within the field of view, and the anterior insulae (Figure 2B).

**Figure 3:**
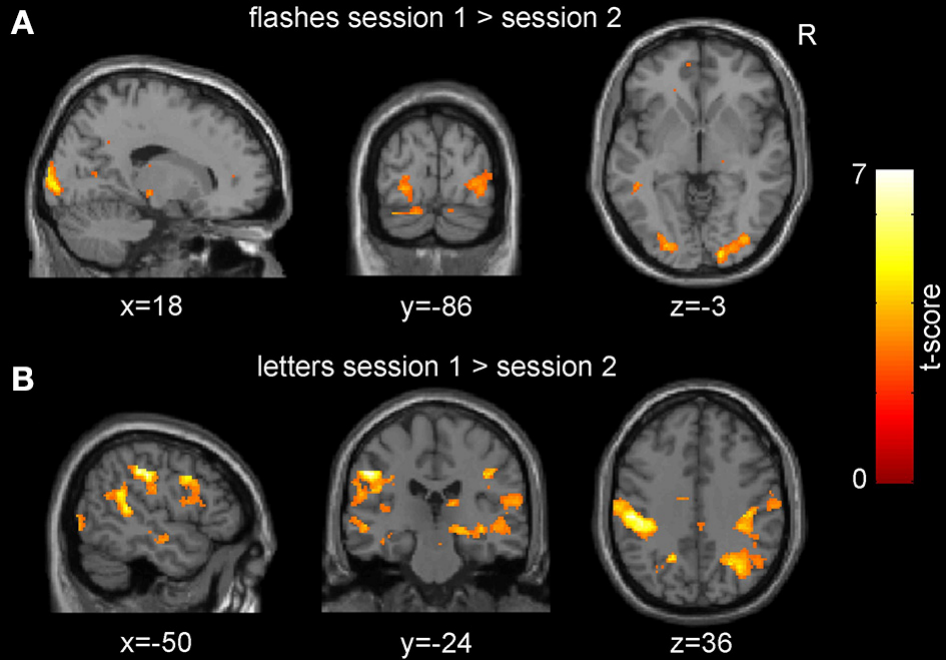
**Group-level session effects**. Neuronal responses in the visual cortex to pattern flashes **(A)** and those to letters and button presses **(B)** were significantly decreased in the post- compared to the pre-high frequency stimulation (HFS) session (*p* < 0.01, uncorrected for multiple comparisons for display purposes).

We observed no significant increases in activations related to low frequency checkerboard stimuli in the second (post-HFS) compared to the first (pre-HFS) session at the multi-subject level (*p* < 0.01 uncorrected for multiple testing at the voxel level and *p* < 0.05 at the cluster level using family-wise error correction). Contrary to that, the inverse contrast revealed a significantly stronger response to the flashes in the pre-HFS compared to the post-HFS session (peak voxel at [18, -96, 2], V1: 100% according to probabilistic atlas; extent 473 voxels; *p*_FWE_ = 0.036; Figure 3A; Table 1). We observed a similar but non-significant effect in the left hemisphere (Figure 3A, Table 1). Also, the responses to the letters were significantly stronger in the first session compared to the second session, with several significant clusters and the maximal effect in the primary somatosensory cortex (peak voxel at [−50, −24, 36], extent 1721 voxels; *p*_FWE_ = 0.00003; Figure 3B). No significant results were observed for the effects of the parametric modulator in the pre- and post-HFS sessions at the group level.

**Table 1:** **Group-level between session effects**.

***p*_FWE_**	**Cluster extent**	**T-score**	**Z-score**	***p*_unc_**	**Coordinates (mm)**			**Cortical region**
					***x***	***y***	***z***	
**FLASHES SESSION 1 > SESSION 2**								
0.036	748	4.80	3.76	0.000	18	−96	2	Primary visual cortex
0.704	252	4.62	3.67	0.000	−34	−84	−22	Cerebellum
1.000	65	3.95	3.28	0.001	−60	−2	−18	Middle temporal gyrus
0.999	80	3.94	3.27	0.001	−46	−40	−4	Middle temporal gyrus (WM)
0.626	277	3.90	3.25	0.001	−16	−48	28	Lobule V, cerebellum (WM)
0.997	94	3.61	3.07	0.001	6	42	−18	Rectal gyrus
0.998	87	3.55	3.03	0.001	24	−52	44	Superior parietal lobule (WM)
0.999	78	3.06	2.69	0.004	10	46	10	Anterior cingulate gyrus (WM)
**FLASHES SESSION 2 > SESSION 1**								
0.991	114	3.49	2.99	0.001	40	10	0	Insular lobe

*Only clusters with an extent exceeding 50 voxels are displayed. The cluster at [18, −96, 2] reveals a significant signal decrease between session 1 (pre-HFS) and session 2 (post-HFS). WM, region lies in white matter/gray matter boundary, the nearest cortical region is indicated*.

**Figure 4:**
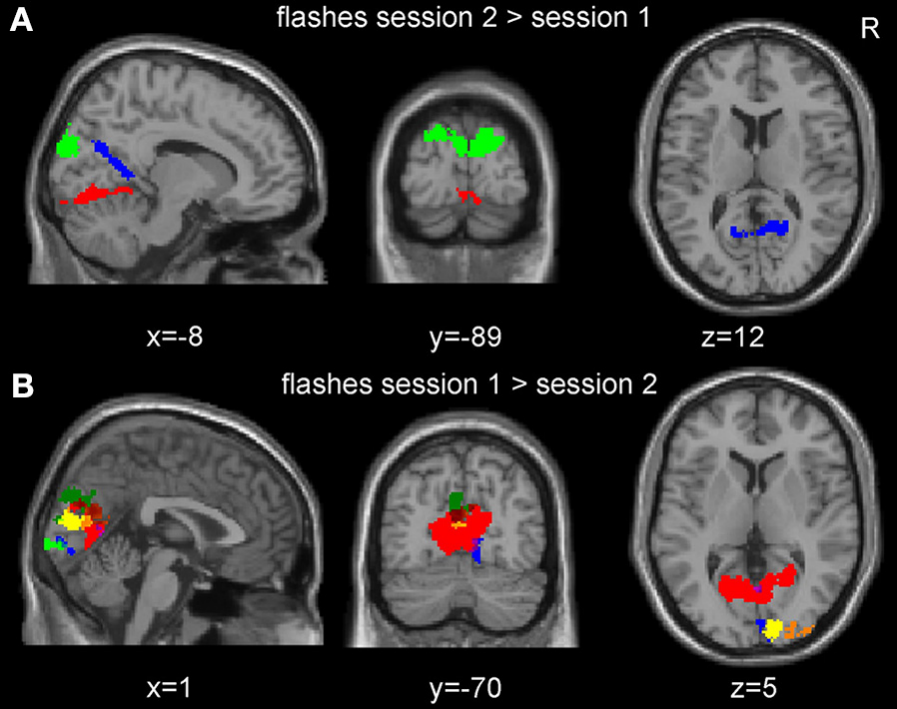
**Session effects of the checkerboard flashes at the single subject level**. **(A)** Overlay of all clusters of significantly increased neuronal responses from the single subject analyses in the post- compared to the pre-high frequency stimulation (HFS) session. **(B)** Overlay of all clusters of significant decreased activation in the post- compared with the pre-HFS session. The clusters from six subjects were partly overlapping. Only clusters within the mask of the visual cortices are displayed here and plotted with a distinct color for each subject (FWE corrected at *p* < 0.05 at the cluster level), for the overlap between the subjects, please refer to Figure S3 (Supplementary material).

In the single subject analyses, responses to pattern flashes were significantly stronger in the pre-HFS compared to the post-HFS session in 6 out of the 18 subjects with partly overlapping clusters (Figure 4B). Only three subjects revealed significant effects for the opposite contrast and clusters were non-overlapping (Figure 4A). The location and extent of areas showing SRM did not depend on the direction of the induced effect (Figure 4 and Supplementary Figure S3).

When testing for linear changes in the response to pattern flashes using a parametric modulator across all 18 subjects and both sessions, two subjects showed a significant linear decrease, while three showed an increase within the mask of the visual cortices. None of the subjects with decreasing activation over time had significantly weaker activations in the post-session. Conversely, one of the subjects with higher activations in the second session also showed a linear increase across both sessions. During the HFS period, the time course of the activation showed no de- or increase over time (see Supplementary Figure S2). Also during the probe sessions response to the checkerboard flashes was not decreasing (see Supplementary Figure S4).

## Discussion

We stimulated our subjects for 120 s with high-frequency (9 Hz) checkerboard flashes in order to induce an increased neuronal response. Contrary to expectations, activations related to pattern flashes, and also to the visual presentation of letters and to button presses were *weaker* after HFS. The typical main effects for pattern flashes serve as a visual localizer (Mohamed et al., 2002; Nelles et al., 2002) and together with the responses to the catch trials found in the current study indicate the technical validity. The accurate button presses in responses to catch trials show that subjects were attentive during the task.

Our findings seem to contradict the available data on potentiation effects induced by HFS and most specifically the observations by Clapp et al. (2005b) that formed the basis for the current study. They reported increased responses to checkerboard-flashes after HFS with a maximum in the secondary visual cortex (V2) contralateral to the stimulation (peak voxel at [±27, −90, 6] with and without flip in x-direction) and a similar effect in the ipsilateral hemisphere (peak voxel at [±30, −87, 24]). We adopted a liberal significance threshold of *p* < 0.01 at the voxel level from Clapp et al. (2005b), followed by a family wise error correction at *p* < 0.05 at the cluster level. This facilitates the detection of large-area activations but reduces localization power (Friston et al., 1996). The locations of effects were very similar in the current study (9 mm apart; peak voxels at [18, −96, 2], peak voxel in V1, cluster spanning V1, V2, and V3), although the left hemisphere cluster in our study was non-significant and had a more caudal peak voxel [−34, −84, −22].

Before listing potential explanations for these apparent contradictions, we discuss changes made to the original design proposed by Clapp and colleagues and motivate them by referring to the existing literature on SRM. Referring to EEG as well as fMRI studies is justified, as blood oxygenation signal detected by fMRI or near infrared spectroscopy (NIRS) is coupled with the EEG signal during checkerboard reversals (Obrig et al., 2002).

In difference to the only existing fMRI study on visual SRM by Clapp et al. (2005b) we (1) stimulated bihemispherically, as we were not interested in side differences and as several EEG studies have shown VEP potentiation using bihemispherical stimulation with checkerboard flashes (Çavuş et al., 2012) or reversals (Normann et al., 2007; Elvsåshagen et al., 2012). (2) Motivated by related work (Normann et al., 2007), we added catch trials during the probe and potentiation periods to monitor attention, thus ensuring subjects attended to the task and focused constantly, as it has been shown that defocus diminishes VEP responses significantly (Pieh et al., 2005). (3) The probe sessions were shorter. Shorter probe sessions may be more susceptible to novelty effects, i.e., larger neuronal responses to the first presentation of the checkerboard stimulus. However, no such effect became apparent when analyzing the time- course averages across all subjects for each session. (4) The interval between pattern flashes during probe sessions was varied and increased on average to optimize the sampling of the resulting hemodynamic response function and thus design efficiency. Effectively, spacing of flashes remained similar to the block design used by Clapp and colleagues but with the added element of catch trials. Shortening the duration and the interval between blocks assures that the induced effects were not truncated in the frequency domain by the high-pass filter. As the amount of signal-loss will depend on the ordering of blocks, spurious between-session or between-condition effects could be induced by different randomizations between pre- and post-HFS sessions.

Concerning the interval between flashes during the probe phase, studies using pattern flashes found that frequencies of 1 Hz (Teyler et al., 2005) or 0.8 Hz (Ross et al., 2008) result (Teyler et al., 2005) or are indirectly shown to result (Ross et al., 2008) in depression, although another study found no indication for depression at 0.85 Hz (Çavuş et al., 2012). The frequencies used in our design were distinctly lower (0.05–0.23 Hz) and were preferred not only because of design efficiency and the contradictions in the existing literature, but also because very low frequency stimulations (i.e., <<1 Hz) have not been found to induce LTD-like effects in slice preparations and in a study with rodents using a similar VEP paradigm (average frequency 0.067 Hz; Clapp et al., 2006). As expected, the parametric modulator did not reveal a significant linear decrease of the flash responses, which would have indicated LTD-like effects, during the *pre-* and *post-*sessions at the group level.

The duration and stimulus frequency of the HFS period remained identical to related studies (Clapp et al., 2005b; Teyler et al., 2005) and we therefore expected a potentiation. Similar to the discrepancies regarding the induction of low-frequency depression effects, existing studies are discordant in respect to potentiation effects induced by HFS. In an electrophysiological study on six healthy volunteers (Teyler et al., 2005), the N1b signal component of the VEP was increased after HFS with checkerboard-flashes. Another electrophysiological study in 22 healthy subjects (Normann et al., 2007) revealed increased amplitudes of the N1 and P1 signal components of the VEP after potentiation with checkerboard reversals. Although all previous studies reported potentiation effects, studies observed these effects at different frequencies. 9-Hz pattern flashes for 2 min induced LTP-like effects in one study (Teyler et al., 2005) while pattern reversals for 10 min at 2 reversals per second (2 rps) did so in the study by Normann et al. (2007). Contrary to the expectations from brain slices, no potentiation was observed using 19 rps stimulation (Normann et al., 2007). Of note, Normann and colleagues used checkerboard reversals rather than flashes. While Marcar et al. (2004) demonstrated that the extent of activation and signal amplitude in the visual cortex was larger for the flashed than for the reversing checkerboard it is unclear if this affects the induced SRM effects. Available data indicates that a long HFS period, especially when stimulated with a relatively high frequency, involves habituation effects that may interfere with potentiation effects. This interpretation would explain why HFS of 10 min with 19 rps did not induce potentiation in a VEP experiment (Normann et al., 2007) and a decreasing fMRI signal during 28 min of flash stimulation at 8 Hz (Lowen et al., 2009). Data from the current study also provides indications that habituation may be the predominant effect as responses to letters were significantly smaller in the post-HFS session.

As we were initially interested in the inter-individual heterogeneity of SRM, we performed a single-subject analysis. Although activations during the HFS period itself indicated stable responses in all subjects (see Supplementary Figure S2), we observed between-session differences not only in respect to the spatial location but also the direction of this effect. Looking at each subject effectively inflates type I errors but the frequency of observed effects is substantially above the 5% threshold expected by chance. It is noteworthy that 3 out of 18 subjects showed significant increases as it indicates that significant SRM effects exist in both directions as shown for related TMS effects (see below). Associations between the location of areas showing SRM and the direction and extent of the effect did not become apparent (see Figure 4 and Supplementary Figure S3). The absence of such an association is congruent with the group level data of our analyses and the one by Clapp et al. (2005b) as both identified an area at the border of V1 and V2, as the main region for SRM effects despite opposite directionality. This area seems to play a critical role in SRM, irrespective of the direction of the effect. In VEP studies, source reconstruction located component C1 of the VEP in the primary visual cortex, while the subsequent components P1 and N1 in V2 or V3, depending on the study (as summarized in Di Russo et al., 2002). The observed potentiations of N1b (Teyler et al., 2005) as well as N1 and P1 (Normann et al., 2007) therefore support a localization of the effect in the V2 region. Given that we neither observed signal changes during the HFS phase (Supplementary Figure S2) nor a significant linear signal change associated with the parametric modulator, we hypothesize that the SRM effect occurs during the break interval. A complex non-linear effect during the probe phase seems a less likely explanation given the low-frequency chosen and because reported signal changes were close to linear (Teyler et al., 2005).

Taken together, our results indicate a high heterogeneity of LTP-like effects in terms of the location but also their direction. This mirrors studies using SRM paradigms in the auditory system that produced incongruent results (Clapp et al., 2005a; Mears and Spencer, 2012). We propose an interaction between LTP-like effects and predominant habituation effects as an explanation for the heterogeneous signal increases in some subjects, but a signal decrease in the majority of the subjects after HFS (Grill-Spector et al., 2006). A similarly high heterogeneity is reported for LTP-like effects using TMS and the paired associative stimulation paradigm (PAS): As in our study, some subjects revealed decreases instead of the hypothesized increases of the motor evoked potential amplitudes (Fratello et al., 2006; Müller-Dahlhaus et al., 2008). Similar effects may have been observed in other PAS studies as authors report a *post-hoc* exclusion of subjects without a response potentiation (Stefan et al., 2004, 2006). For TMS induced LTP-like effects, heterogeneity was partly explained by age (Müller-Dahlhaus et al., 2008), genetics (Cheeran et al., 2008), daytime (Sale et al., 2007), and attention (Stefan et al., 2004).

However, a recent study applied PAS-TMS, Theta burst stimulation and anodal dDCS and found very few subjects homogeneously responding to all potentially LTP-inducing interventions (López-Alonso et al., 2014). As this is a within-subject study, the impact of factors such as gender, age, and genetics is reduced as a source of variability.

A better understanding of factors leading to a high variability of LTP-like effects, not observed in LTP at the cellular level, is essential to compare different LTP-like effects, to identify the underlying mechanisms and to establish tools to quantify individual synaptic plasticity *in-vivo*.

### Conflict of interest statement

The authors declare that the research was conducted in the absence of any commercial or financial relationships that could be construed as a potential conflict of interest.
