# Supplementary material for: Heterogeneity of stimulus-specific response modification—an fMRI study on neuroplasticity
Source: Front Hum Neurosci. 2014 Sep 8;8:695. doi: 10.3389/fnhum.2014.00695 (PMC4157554; doi:10.3389/fnhum.2014.00695)
Supplement: Supplementary file 1 [file DataSheet1.PDF]

Supplementary figures

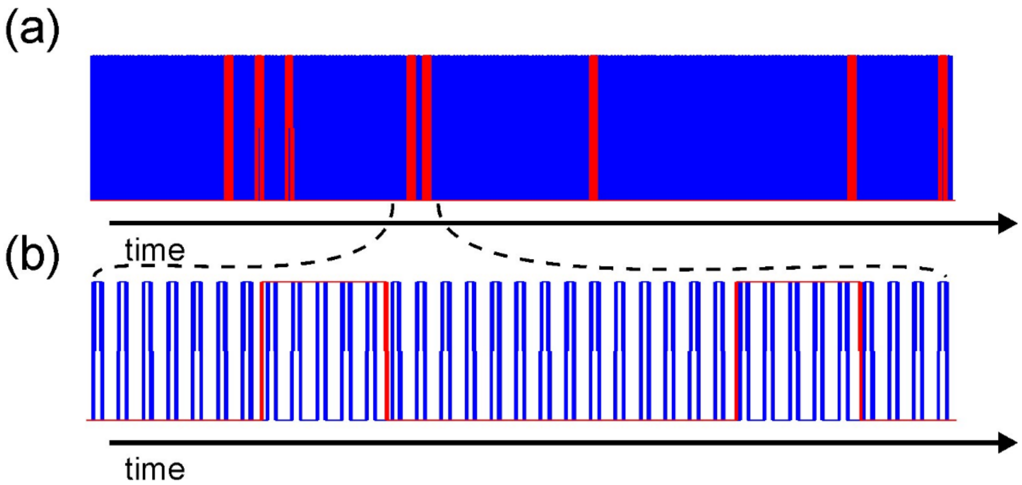

Fig. S1: (a) timing of the checkerboard-flashes (blue) and the letters (red) during the high-frequency stimulation period. In (b), a cutout of (a) is displayed for better visualization of the individual flashes and letters. Each checkerboard-flash was presented for 33ms, while each letter was presented for a duration of 555ms.

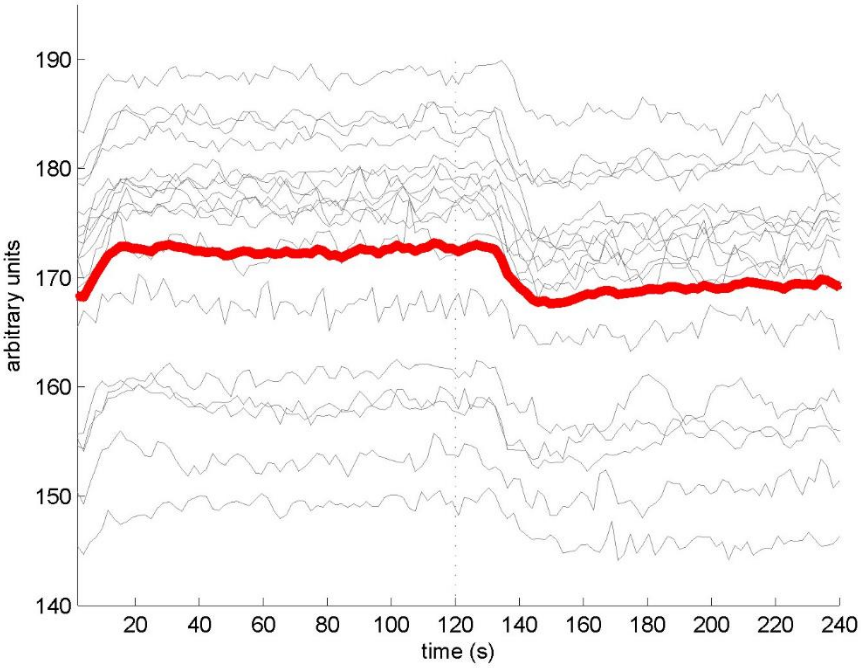

Fig. S2: Time course of the BOLD signal in the peak coordinate of the checkerboard flashes (x,y,z: [12, -94, 2]) during the high-frequency stimulation period (0-120 s) and the subsequent rest (121-240 s). The grey lines represent the time course of the individual subjects, and the bold red line represents the mean time course over all subjects.

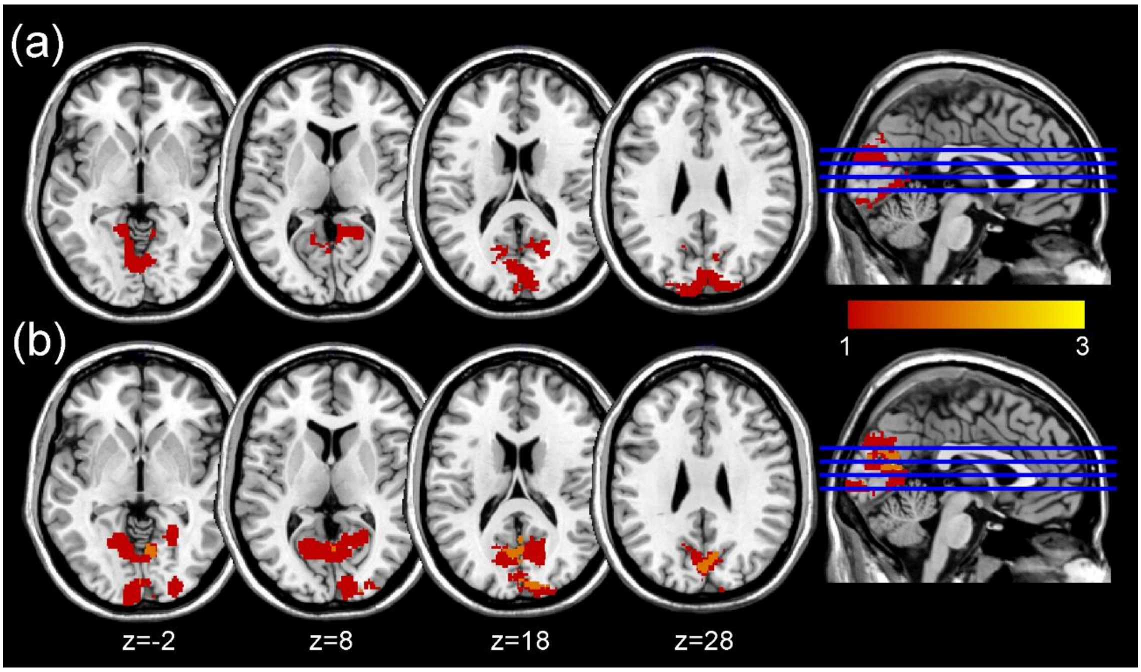

Fig. S3: Overlay of all clusters with significant signal increases (a) and decreases (b) of the individual subjects. Only clusters within the mask of the visual cortices are displayed here. Color encodes the number of significant clusters at a distinct voxel, red indicating one cluster at a voxel, and orange and yellow indicating the overlap of two, respective three clusters (FWE corrected at $p<0.05$ at the cluster level).

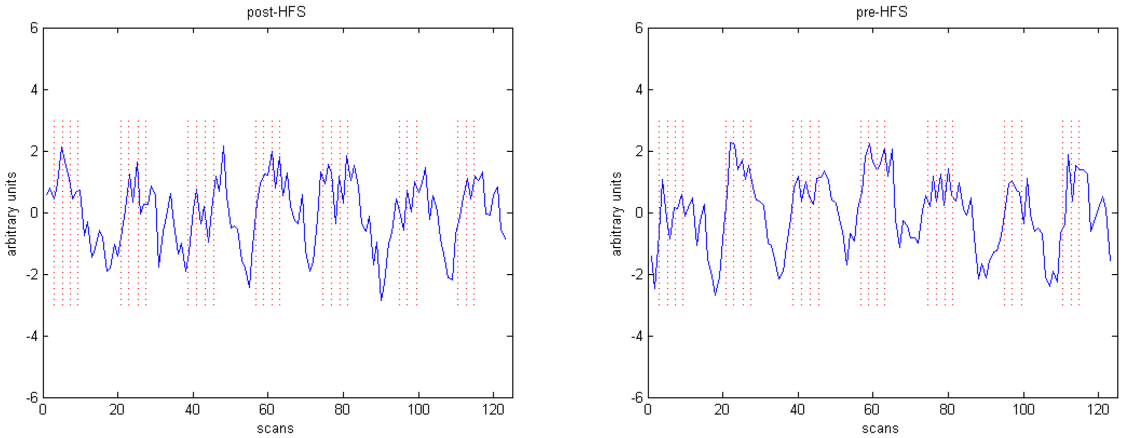

Fig. S4: BOLD time-course during the probe sessions. The blue line indicates the mean BOLD-signal over all subjects in a sphere (radius: 8 mm) around the peak voxel obtained from the main effect of flashes (x,y,z: [12, -94, 2]). Each dotted red line indicates the presentation of a checkerboard flash.
